# Simulated trapping and trawling exert similar selection on fish morphology

**DOI:** 10.1002/ece3.8596

**Published:** 2022-02-12

**Authors:** Davide Thambithurai, Anita Rácz, Jan Lindström, Kevin J. Parsons, Shaun S. Killen

**Affiliations:** ^1^ 54616 Institute of Biodiversity, Animal Health and Comparative Medicine University of Glasgow Glasgow UK; ^2^ Department of Genetics ELTE Eötvös Loránd University Budapest Hungary

**Keywords:** FIE, fishing, geometric morphometrics, human impact, locomotion, morphology

## Abstract

Commercial fishery harvest can influence the evolution of wild fish populations. Our knowledge of selection on morphology is however limited, with most previous studies focusing on body size, age, and maturation. Within species, variation in morphology can influence locomotor ability, possibly making some individuals more vulnerable to capture by fishing gears. Additionally, selection on morphology has the potential to influence other foraging, behavioral, and life‐history related traits. Here we carried out simulated fishing using two types of gears: a trawl (an active gear) and a trap (a passive gear), to assess morphological trait‐based selection in relation to capture vulnerability. Using geometric morphometrics, we assessed differences in shape between high and low vulnerability fish, showing that high vulnerability individuals display shallower body shapes regardless of gear type. For trawling, low vulnerability fish displayed morphological characteristics that may be associated with higher burst‐swimming, including a larger caudal region and narrower head, similar to evolutionary responses seen in fish populations responding to natural predation. Taken together, these results suggest that divergent selection can lead to phenotypic differences in harvested fish populations.

## Graphical abstract

We conducted work assessing how simulated trapping and trawling selects for specific fish morphologies. We quantified selection by using geometric morphometrics. Results indicate that both gears exert similar selection pressure on morphology.

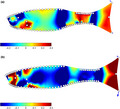

## 1 INTRODUCTION

The selective harvest of wild animals by humans is a powerful evolutionary force with the potential to affect population resilience and ecological dynamics (Allendorf & Hard, 2009; Darimont et al., 2015; Palkovacs et al., 2012; Palumbi, 2001). Fisheries, owing to their high rate of exploitation and inherent selectivity, can be particularly strong drivers of evolutionary change (Darimont et al., 2015; Fugère & Hendry, 2018; Heino et al., 2015). Phenotypic selection in fisheries has been linked to many traits including those associated with behavior (Diaz Pauli & Sih, 2017; Thambithurai et al., 2018), habitat preference (Arnason et al., 2009), and maturation timing (Feiner et al., 2015). Size‐selective harvesting is particularly prevalent, as physical constraints imposed by net mesh sizes and other factors can remove the largest individuals from a population, thereby decreasing the average size (Fugère & Hendry, 2018; Swain et al., 2007; Uusi‐Heikkilä et al., 2015). Notably, however, studies on harvest selection have tended to focus on size *per se* as the trait of interest, while studies on morphological traits are rarer (Alós et al., 2014; Hamon et al., 2000). Organismal morphology plays a significant functional role in ecology, influencing resource utilization, behavior, reproduction, and habitat use (Wainwright & Reilly, 1994). Importantly, the heritability of morphological traits is generally higher than that of behavioral or physiological traits (Carlson & Seamons, 2008; Mousseau & Roff, 1987), making morphology more likely to evolve in response to selection.

Morphological adaptations stemming from predator avoidance are ubiquitous in natural prey populations, and often linked to functional performance during escape responses or sensory capacity (Oufiero et al., 2011; Wilson et al., 2005). For example, prey fish that co‐occur with predators often exhibit morphological characteristics that allow them to employ specific swimming tactics that maximize their chances of escape; these morphologies include fusiform body shape, longer caudal peduncles, and larger fins (Ghalambor et al., 2004; Ingley et al., 2014). For fishes, swimming is of critical importance to predator escape and can be split into two primary modes: sustained and unsustained swimming. Sustained swimming comprises aerobic locomotion that is common during foraging, exploration, and habitat choice, and it excludes high speed bursts. Unsustained swimming includes anaerobic locomotion in the form of fast‐starts (a reflexive and rapid escape response) and burst‐type accelerations, and is commonly employed during predator–prey encounters (Domenici & Blake, 1997; Langerhans, 2009a; Videler, 1993). Typically, within species, morphologies associated with these two locomotory modes broadly fall into two categories: sustained swimming is enhanced by straighter bodies that are more streamlined and less deep, while unsustained performance capacity is reflected by greater body depth, especially in the posterior portion of the body (Langerhans & David, 2010; Skúlason et al., 2019; Webb, 1984). Interindividual differences in shape can therefore dictate the functional performance of fish within a population (Kern & Langerhans, 2018). Across species, the relationship between body shape and swimming style is more complicated, with some fish families displaying a straight elongated body but specializing in unsustained swimming (e.g., *Esocidae*). Interindividual variation in morphology within a population means that patterns of morphological adaptation can be predator‐specific (e.g., Heynen et al., 2017), and also heavily shaped by other environment‐specific selection gradients (Burns et al., 2009). Many of the traits promoted by selection from natural predators may also be targets of anthropogenic selection (Hollins et al., 2018; Langerhans, 2009b), therefore evolutionary pressures in some cases may coincide or diverge. While selection may shift the mean morphology of a population, it can also reduce levels of phenotypic disparity, as specific morphological features are favored.

Different fishing gear types intrinsically differ in the way they capture fish. Some gears are described as relatively active, while others are considered passive (Hollins et al., 2018). An active fishing method is more analogous to a pursuit predator, while a passive method is more akin to a sit‐and‐wait type predator (He, 2010). The selective forces stemming from these different types of gear can vary, perhaps promoting divergence in the morphology and behavior of targeted fish. For instance, slower swimming fish may fail to escape from an oncoming trawl net (an active gear) as they are outpaced, leading to positive selection on faster swimmers. Although sustained swimming is not typically employed during natural predator encounters, it might be more commonly used in escape from trawl nets (Bayse et al., 2016), as cruising in front of a net might be advantageous to fish trying to escape (i.e., maximizing the time they have to take evasive maneuvers), this could lead to positive selection on more sustained swimming phenotypes within the population. Maneuverability itself could also potentially be favored in fish trying to escape trawl nets, as individuals with higher burst‐swimming capacity may be able to carry out more extreme maneuvers at faster speeds thus decreasing their vulnerability to trawling (Killen et al., 2015). In contrast, trapping, which is a passive fishing method, might be less selective for locomotor capacity because capture is dependent on fish finding and voluntarily entering a trap. However, specific morphological traits such as increased body depth (indicative of condition or foraging ability) might reduce the probability of capture if it limits the ability to enter or exit traps. Additionally, morphology might also be correlated to internal states such as energy stores and hunger levels, thereby altering vulnerability to capture (Härkönen et al., 2014).

Laboratory approaches offer an opportunity to investigate the potential morphological selection that may occur in fisheries through experimental manipulations that are not possible at a broader scale. Here we used zebrafish (*Danio rerio*), a gregarious benthopelagic species, with a broadly similar structural morphology to some commercially fished species (e.g., *Gadus morhua*) to: (1) determine if trapping and trawling select on specific morphologies; (2) assess how the morphologies they select on differ; and (3) identify specific morphological components associated with higher vulnerability to capture for each gear. To gain a general understanding of the strength of selection on shape, we calculated morphological disparity to discern the relative degree of variability between, and within, fish categorized by vulnerability. We also calculated caudal fin ratios to identify whether these had a significant impact on vulnerability to trawling and trapping, given that they are known correlates of activity (Plaut, 2000). To address these aims we developed small‐scale trawling and trapping simulations. We predicted that individuals with morphological characteristics associated with better swimming performance would be less vulnerable to capture by trawling. For trapping, we predicted that narrower fish would be more vulnerable to capture as they would be able to enter traps more readily.

## 2 METHODS

### 2.1 Study organisms

Adult zebrafish (~6 months of age) were sourced from rearing ponds in Singapore during spring 2017 (JMC Aquatics, Sheffield). Following arrival, zebrafish were maintained in 300 L glass stock tanks held under a constant 13h:11h light‐dark (white fluorescent light) cycle and supplied with dechlorinated and continuously filtered water (mean ± range = 26 ± 1°C), and fish density was kept below 5 fish L^−1^. Fish were fed twice daily *ad libitum* with a combination of commercial feed (TetraMin 115 (flake); and ZM small granular (pellet)) and live 48 h hatched *Artemia nauplii* (Sanders Great Salt Lake Artemia Cysts) (full husbandry protocols can be accessed here: dx.doi.org/10.17504/protocols.io.bqf9mtr6; accessed date: December 1, 2020).

## 3 SUMMARY OF EXPERIMENT

To test whether trapping or trawling (active = trawling; passive = trapping) can select on fish shape morphology, we subjected zebrafish to repeated assays that were used to create vulnerability distributions for each gear type. Fish (*n* = 510) were housed in four identical aquaria, and each group (trap or trawl) was held simultaneously under the same husbandry conditions described above. A month before the start of the experiment, fish were tagged using visual implant elastomer (VIE) (Northwest Marine Technology, WA, USA) in four dorsal tag locations with an individual code identifier (Rácz et al., 2021). Wet mass and standard length were measured for each individual (Table S1), and each fish was also sexed based on a combination of color and morphology. A few days prior to each trial, fish were moved to a zebrafish rack system (Z‐ Hab system, MBK Ltd, Nottingham, UK) and kept at densities below 6 fish L^−1^. All animal experimental procedures were executed in accordance with the UK's Home Office guidelines (Project Licence no. 60/4461, approved 2015).

### 3.1 Trawling

#### 3.1.1 Trawl setup

Trawl trials started in January 2018. All simulations were performed in a temperature‐controlled (mean ± SD = 26 ± 0.3°C) 90‐l Steffensen‐type swim tunnel (Loligo systems, Tjele, Denmark). A small‐scale model of a commercial trawl (modified from a small trawl constructed at the Marine Institute, St. John's, NL, Canada) was fitted to the working section of the flume (66 cm L × 20 cm W × 20 cm H) using plastic inserts. Mesh size graduated from 9 mm at the mouth to 1 mm toward the tail. A stainless‐steel frame was used to fix the net to the bottom of the flume and to keep the net mouth consistent during trials. The net frame covered the entire cross‐section of the flume, with the exception of two escape holes (each 9 cm^2^) at the top left and right of the frame. Fish could also escape under small gaps beneath the lead line during periods of elevated flow. This setup emulated some of the escape opportunities afforded to fish in actual trawling scenarios. To minimize stress on captured fish, and accurately monitor captures, the end of the net (codend) was fitted with a wedge‐shaped fish retention chamber made from transparent acrylic sheeting that minimized hydraulic flow and turbulence. Lighting during the experiment was provided by two 44w LED tube lights. A single camera (GoPro Hero 4, San Mateo, California, USA) mounted over the retention area of the net was used to monitor captures. This setup, together with the unique tags of each fish, allowed an accurate time for each capture to be recorded. To reduce external disturbance a black plastic sheet was hung around the flume throughout the trial.

#### 3.1.2 Test procedure trawling

**FIGURE 1:**
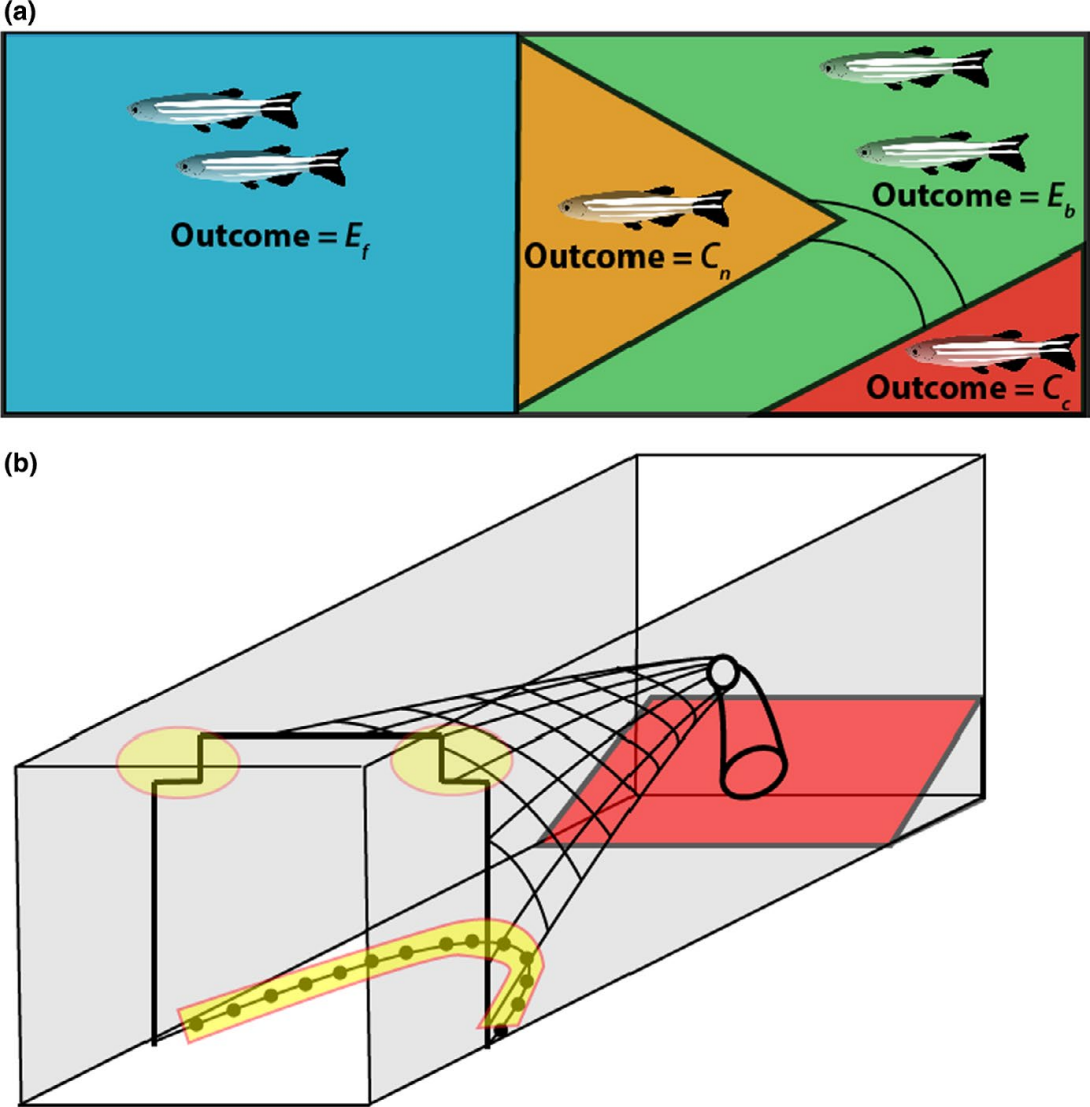
Simplified scheme of trawl setup. (a) Scoring applied to the trawl trials as seen in profile: *E*_B_ = fish escaped beyond the net; *E*_F_ = fish escaped in front of the net; *C*_N_ = fish were caught within the net but did not enter the retention area (cod‐end); *C*_C_ = fish were caught in the retention area. The trawl apparatus was fitted to the working area of a recirculating flume (b). Areas shaded in yellow indicate potential escape routes, fish were only able to escape under the footrope when turbulent flow lifted it momentarily. For more details, please see supplementary materials (Figure S2)

Each fish completed three trawl trials. Fish were tested in groups of 10, with individuals haphazardly netted from their holding tanks to each group prior to testing. Owing to some mortality prior to the start of the experiment, one trial per day had between 7 and 9 fish per group. We are confident that this did not influence the results given the high number of replicates and fish tested. Test order was randomized among individuals, and a minimum rest time of 48 h was maintained between each trial for each fish. Fish were fasted 24 h prior to testing and fed immediately after the trials. At the beginning of each trial, fish were netted from the rack system and transferred to the acclimation section of the flume. Fish were acclimated to the flume for 20 min at <10 cm s^−1^, allowing fish to orient against the current and swim slowly but steadily. Following this period, the screen at the rear of the acclimation area was removed and over the next minute the water speed within the flume was increased to the target water velocity for that trial. We chose the lowest speed which stimulated burst‐type swimming in all fish within the flume, analogous to what occurs in an actual trawl fishery (He, 2010). To account for training effects arising from repeated testing, maximum water speed was increased slightly with each trial: the first replicate was conducted at 56 cm s^−1^, the next at 58 cm s^−1^, and the final trial was conducted at 60 cm s^−1^. Each trial lasted for 10 min, and at the end of this period water speed was decreased back to <10 cm s^−1^ and the acclimation screen was re‐introduced to the arena; this effectively prevented any fish escaping the net, but also split the working area into four distinct sections used in creating a vulnerability index (Figure 1; Figure S2).

#### 3.1.3 Trawling vulnerability index

At the end of each trial, all fish in the flume were scored a value according to their end location, the scores were based on a most favorable (least vulnerable) to least favorable outcome (most vulnerable): ***E*_B_** = fish escaped beyond the net; ***E*_F_** = fish escaped in front of the net; ***C*_N_** = fish were caught within the net but did not enter the retention area; ***C*_C_** = fish were caught in the retention area (Figure 1). Videos were analyzed to assess: (i) the time taken for a fish to be captured (i.e., enter the cod end); (ii) the number of fish entering the cod end (iii); and the order of fish entering the cod end. Only fish entering the retention area (score = ***C*_C_**) were given a capture time for that replicate. We chose to use the above scoring based on the following: ***E*_B_** is considered as the most preferential score, as fish that pass the net posteriorly and aren't caught in the retention area have “fully escaped,” and do not run the risk of being recaptured; ***E*_F_** is considered as the second most beneficial outcome, as through either performance or behavior the individuals that attained this score remained in front of the net and weren't captured, nevertheless were still in a potentially “catchable population”; ***C*_N_** is considered as the second to worst outcome as the fish are in the net and likely would have ended up in the retention area with time, additionally escape from here is considered more difficult than for fish in front of the net and any fish within the net itself is more likely to be captured as a net is pulled to the surface; and finally, ***C*_C_** is considered the most deleterious outcome as there is little opportunity for escape once in the retention area. To obtain a vulnerability index for trawling, a series of user made functions were developed in R version 3.5.3 (R Core Team, 2017). A single vulnerability index was calculated for each fish based on three scores attained over all trawl replicates.

To increase the resolution of the vulnerability distribution we introduced a series of penalties based on the scores achieved by each fish in each trial as follows: a score of ***E*_B_** = 50, ***E*_F_** = 100, ***C*_N_** = 200, and ***C*_C_** = 300. In addition to the nominal 300 achieved for a ***C*_C_** score, an additional time penalty was added to the total (600 s ‐ time of entry seconds). In equation form (Equation 1), for each fish, this is represented as:

(1) $$V\;=\sum_{i=1}^{3}y_{i}+x_{i}$$

where V is the vulnerability index over three trials, $y_{i}$ is the general penalty score achieved during each trial, and $x_{i}$ is a time penalty dependent on whether a fish achieved a ***C*_C_** score for each trial. Thus, a potential maximum achievable score over the duration of the three trials for an individual fish was 2700 ([***C*_C_** + 600] + [***C*_C_** + 600] + [***C*_C_** + 600]) and a minimum of 150 (***E*_B_** + ***E*_B_** + ***E*_B_**).

Once all fish were assigned an individual vulnerability based on all trawl replicates, the top 25% and bottom 25% of the distribution were identified as the most and least vulnerable. This process was repeated across both trawl tanks.

### 3.2 Trapping

#### 3.2.1 Trap setup

Trap trials were conducted in a glass aquarium (122 cm L × 60 cm W), filled to a depth of 20 cm with temperature‐controlled water (mean ± SD = 26 ± 0.3°C). The aquarium floor was covered in sand and three artificial plants were used to provide landmarks and a more natural environment. A transparent chamber (30 cm L × 25 cm W × 25 cm H) attached to the side of the arena and equipped with a pulley system that allowed a vertical door to be lifted was used to acclimate fish prior to the start of each trial. Across all trials, three custom‐made scaled replica finfish traps were used (Video S2) for trapping; the bottom net portion of the traps was excluded from the original design to allow easy removal of fish. Traps consisted of a brass frame covered in white netting (<1.5 mm mesh size) with two inverted funnel entrances measuring ~5 cm^2^. Prior to each trial traps were baited with commercial fish pellets (6 mm Goldfish pellets, Vitalis Aquatic Nutrition, Doncaster, UK). The location of both traps and plants was randomized before the start of each trial by dividing the trap area in a grid and using a random number generator to choose the location of each trap/plant for that trial. At all times traps were placed a minimum of 10 cm from the walls of the tank to ensure unobstructed entry. Just as in commercial finfish traps, fish were allowed to freely enter or exit throughout the duration of the trial. The trial arena was covered with dark plastic sheeting to minimize external disturbance and to provide a uniform background for the camera which was placed to offer a side view the aquarium (Logitech HD Webcam c920; Logitech Europe S.A., Lausanne, Switzerland).

#### 3.2.2 Test procedure trapping

Prior to commencing each trial a clear screen was slid into place separating the acclimation chamber from the rest of the arena. Following this, 20 fish were netted haphazardly from the zebrafish rack system, inserted in a holding container, and transferred to the acclimation chamber of the arena; the overall netting procedure took <2 s, and during this time, fish were exposed to air. To obtain accurate visualizations of fish behavior we started video recording at this point. Fish were left to acclimate for 20 min after which the screen separating the acclimation chamber from the main section of the arena was lifted remotely using a pulley system. Fish were then allowed 30 min to interact with the traps. At the end of each trial an observer immediately covered each of the three traps with perforated plastic containers, and this effectively stopped any ingress/egress from the traps and allowed for an accurate count of fish both within and outside the traps. Each fish completed a total of three trap trials. Similar to trawling, test order was randomized among individuals and a minimum rest time of 48 h was maintained between each trial for each fish. Fish were fasted 24 h prior to testing and fed immediately after the trials.

#### 3.2.3 Trapping vulnerability index

Similar to trawling trials, a series of user made R functions were used to rank fish according to trap vulnerability. Fish that were captured were scored depending on the trial in which they were caught (**3** = trial 1; **2** = trial 2; and **1** = trial 3), and if not captured, a fish would receive a zero score. A capture was defined as a fish being trapped at the end of the trial. Thus, the maximum achievable score for a single fish using this method across all replicates would be six, with a six being most vulnerable and zero being least vulnerable. These penalties were used to obtain higher resolution than would be achievable using a binary score. We decided to weight earlier trials more to account for potential learned behavior in each subsequent trial, and this is in contrast to a wild fishery where individuals be removed from the population, unless discarded and re‐captured. As for trawling, the top 25% and bottom 25% of the vulnerability distribution using the above scoring systems were used to create the high vulnerability and low vulnerability groups.

#### 3.2.4 Image analysis and morphological landmarking

**FIGURE 2:**
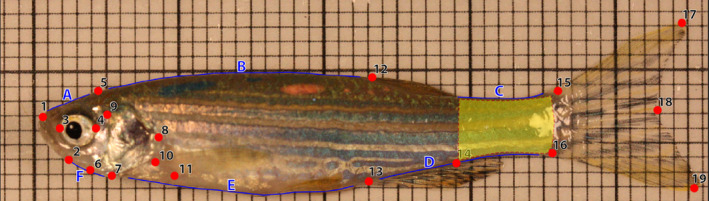
Numbered landmarks (red points) represent the following features: 1 anterior tip of snout, 2 posterior tip of lower mandible, 3 and 4 anterior and posterior middle axis of the eye, 5 occiput, 6 anterior tip of opercular bone, 7 inferior edge of opercular bone, 8 point of maximal exertion of operculum, 9 antedorsal origin of opercle, 10 superior insertion of pectoral fin, 11 inferior insertion of pectoral fin, 12 anterior insertion of dorsal fin, 13 anterior insertion of the anal fin, 14 posterior insertion of the anal fin, 15 superior insertion of the caudal fin, 16 inferior insertion of the caudal fin, 17 superior tip of the caudal fin, 18 posterior central edge of the caudal fin, 19 inferior tip of the caudal fin. A number of semi‐landmarks were used, and these were placed between the fixed landmarks: (a) between landmarks 1 and 5 (*n* = 8); (b) between landmarks 5 and 12 (*n* = 15); (c) between the posterior edge of the dorsal fin and 15 (*n* = 10); (d), between 13 and 16 (*n* = 14); (e), between 7 and 13 (*n* = 17); f, between 2 and 7 (*n* = 8). The area shaded in yellow is indicative of the caudal peduncle in zebrafish

Following behavioral assays all fish were photographed on top of laminated graph paper (to provide a scale) using a digital camera (Canon EOS 450 D, Canon, Tokyo, Japan) mounted with a macro lens (Canon EFS 60 mm, Canon, Tokyo, Japan). Using the vulnerability criteria discussed above we chose 111 fish from the trawl group (Trawl/high = 54; Trawl/low = 57) and 107 fish from the trap group (Trap/high = 55; Trap/low = 52) for further analysis. We used imageJ (version 1.52a) to manually measure the height (lower to upper tip) and area of the caudal fin for all fish in order to calculate caudal fin ratios. Coordinates of morphological landmarks were acquired from two‐dimensional lateral photographs of live individuals using tpsDig2 (Rohlf, 2013). We digitized 19 homologous landmarks (LMs) and 72 semi‐landmarks (semi‐LM) (Figure 2) and a scale for each individual. We chose the landmarks based on previous literature that has assessed shape change in response to natural predation and anthropogenic harvest (Alós et al., 2014; Domenici et al., 2008; Langerhans et al., 2004), as well as expert knowledge.

#### 3.2.5 Analysis

All statistical analyses were performed in R 4.1.2 (R Core Team, 2017), a list of packages used for the main analyses are provided in the supplementary materials (Dryad Digital Repository: https://doi.org/10.5061/dryad.pk0p2ngq2). Geometric morphometric analyses were conducted using the package “geomorph” (version 3.0.7) (Adams & Otárola‐Castillo, 2013). First, all standard landmarks were transformed into shape variables (Procrustes coordinates) by generalized least squares Procrustes superimposition which translates, rotates, and adjusts the landmarks across specimens to a common scale (Zelditch et al., 2004). We then defined sliding landmarks to identify particular curved features of interest (Bookstein, 1997), and using the *define*.*sliders* function we added them to the dataset. To test for allometry (the dependence of shape on size), we used a multivariate regression of Procrustes coordinates on centroid size using the *procD*.*allometry*() function on fish from each gear type. Statistical significance was assessed using 10,000 permutations.

Morphological divergence in shape between high and low vulnerability fish across gear types was tested for using a Procrustes ANOVA with a design analogous to a global multivariate analysis of covariance (MANCOVA), and a type I sums of squares and cross‐product computation was used. The model included Procrustes shape coordinates as dependent variables, centroid size as a covariate (to minimize allometry), and vulnerability (high vulnerability or low vulnerability), sex and gear as explanatory variables. We also included interaction terms between vulnerability and sex to assess whether any differences in shape within vulnerability groups differed due to sexual dimorphism, as well as an interaction between gear and vulnerability to test whether gear type influenced what type of shape phenotype was vulnerable. Significance for the model was assessed with 10,000 iterations.

Procrustes shape coordinates were also analyzed with a multivariate linear discriminant analysis (LDA). This technique is a supervised machine learning method that classifies fish into high and low groups given their shape. We firstly obtained size‐independent variables for the LDA using two regressions, for trawl and trap fish respectively, using Procrustes shape coordinates as dependent variables and Centroid size as a covariate. Size‐independent coordinate variables (residuals from the models) were extracted, normalized, and then used as independent variables in each LDA for both gears. For each gear, data were split into training (70% of individuals) and test datasets (30% of individuals), the algorithm was then used on the training dataset, and the performance of the LDA was quantified by comparing results to the test dataset.

To visualize localized shape differences among groups, we used MATLAB (version R2009B, Mathwork) and the package Lory (version 1.0) (Márquez et al., 2012) which transforms shapes into functions that are then used to inform local deformations. This allowed for an estimation of differences between fish beyond that which is typically afforded by deformation grids. For each gear type (trap and trawl) and sex, we plotted the average shape of high versus low vulnerability fish.

We also measured caudal fin height and area using imageJ, which allowed us to calculate caudal aspect ratios for each fish and have a more quantitative measure of how tail morphology relates to fishing vulnerability (Sambilay, 1990). We used both caudal aspect ratio and caudal fin area as response variables in two linear regression models with standard length, vulnerability (high vulnerability and low vulnerability), sex, and gear as explanatory variables. Again, we included interaction terms between vulnerability and sex, as well as vulnerability and gear type. Model selection was conducted via Akaike's Information Criteria (AIC), with only the most parsimonious models being selected (Burnham et al., 2011).

We investigated the degree of morphological disparity among the high vulnerability and low vulnerability groups. This allowed us to make a more quantitative interpretation on the level of pairwise variation in shape. Shape disparity (i.e., the multivariate variance in morphospace) was calculated using Procrustes shape variables, and pairwise comparisons between gears (trawl and trap) and sexes were calculated using the function *morphol*.*disparity* from the package geomorph. The number of iterations for the permutation tests was set to 10,000. This function estimates Procrustes variance by dividing the sum of the diagonal elements of the group covariance matrix by the number of observations in the group (Zelditch et al., 2004).

Lastly, to investigate the relationship between body condition and vulnerability, we calculated the scaled mass index (SMI) for each fish by calculating ${\hat{M}}_{i}$ the predicted body mass (scaled to $L_{0}$) for each individual i (Peig & Green, 2009):

$${\hat{M}}_{i}=M_{i}{\left[\frac{L_{0}}{L_{i}}\right]}^{b_{\mathrm{SMA}}}$$

where $M_{i}$ and $L_{i}$ are the body mass and standard length of individual i, respectively; $b_{\mathrm{SMA}}$ is the slope of standardized major axis regression of log_n_ mass and log_n_ length. $L_{0}$ is the arithmetic mean value of length. We used SMI in a linear regression as a response variable and included vulnerability, sex, and gear type as categorical explanatory variables to assess the relationship between condition and vulnerability across both gear types. We tested for the interactive effects of sex and gear on SMI, and selected the best fitting model from comparison of AIC values.

## 4 RESULTS

**FIGURE 3:**
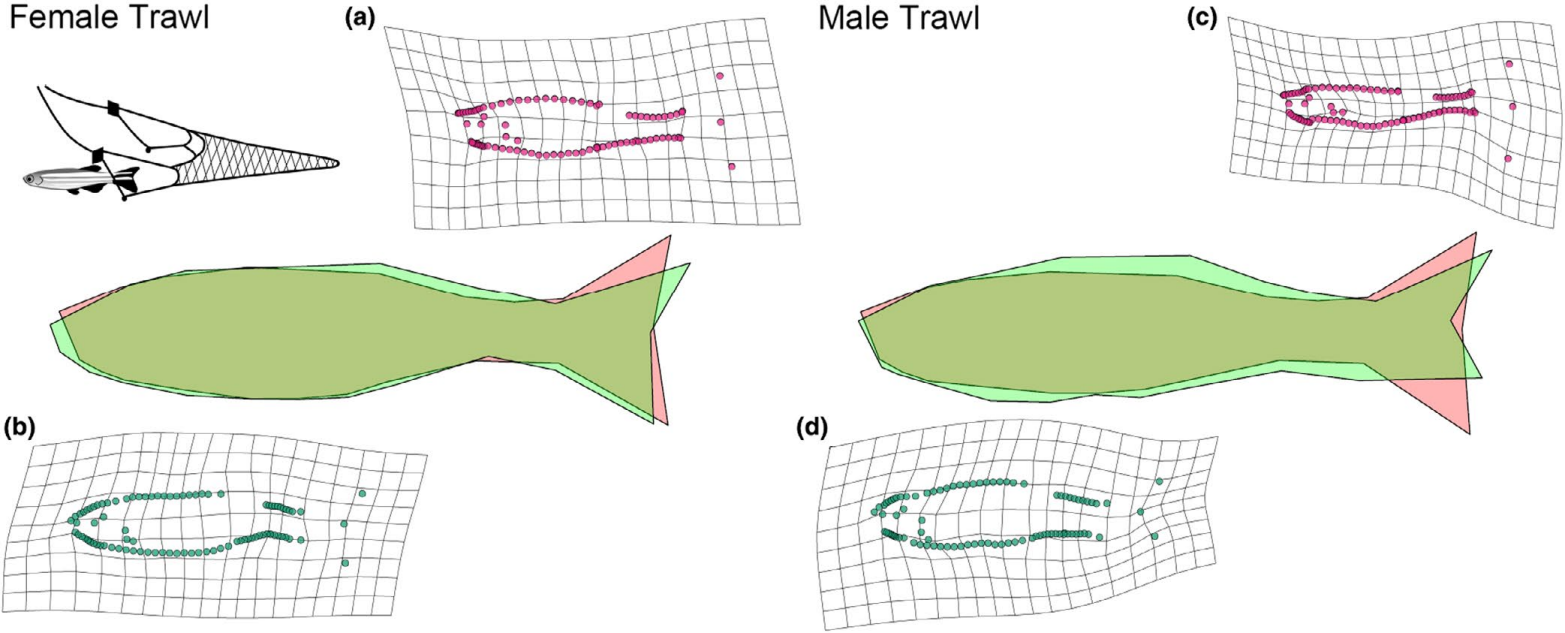
Visualization of body shape variation between high and low vulnerability trawl fish for females and males. Polygon outlines (of LMs) representative of low vulnerability fish (green) against high vulnerability fish (red) are shown in the middle (magnified to 7). Thin‐plate spline transformation grids (magnification set at 7) of the average Procrustes adjusted shape for high vulnerability (a, c) and low vulnerability (b, d) for each sex are also shown

**FIGURE 4:**
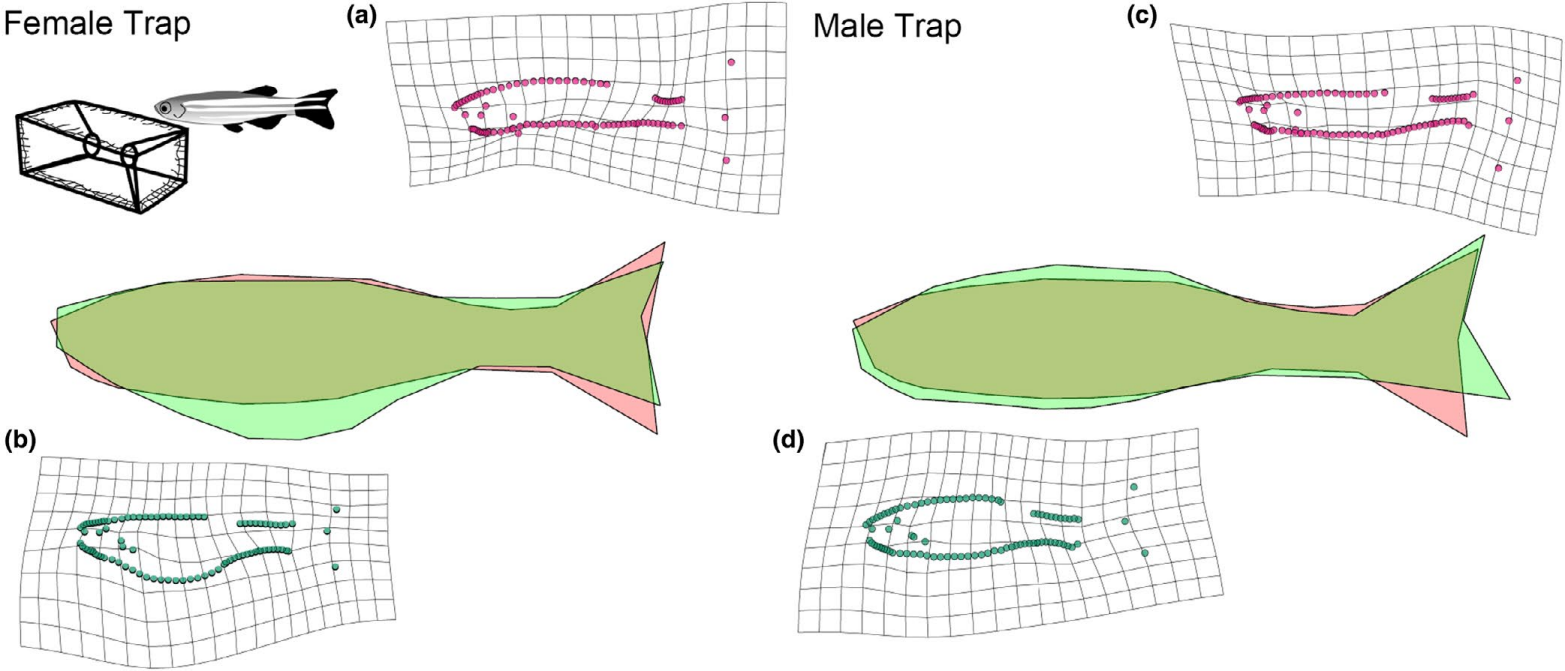
Visualization of body shape variation between high and low vulnerability trap fish for females and males. Polygon outlines (of LMs) representative of low vulnerability fish (green) against high vulnerability fish (red) are shown in the middle (magnified to 7). Thin‐plate spline transformation grids (magnification set at 7) of the average Procrustes adjusted shape for high vulnerability (a, c) and low vulnerability (b, d) for each sex are also shown

High vulnerability fish were morphologically different from low vulnerability fish, but these differences did not differ between gears (Table 1). Fish that were more easily captured were generally shallower bodied (Figures 3 and 4), with vulnerability accounting for approximately 1% of morphological variation (% of shape variance attributable to vulnerability in the MANCOVA). The interaction term between sex and vulnerability was not significant, indicating that shape in high and low vulnerability groups did not differ based on sex. We found allometry in morphology to be present in both trawl fish (df = 109, *F* = 4.7, *p* < .001) and trap fish (df = 105, *F* = 8.4, *p* < .001).

**TABLE 1:** Results of Procrustes ANOVA testing body shape differences between high and low vulnerabilities across gear type

Term	Estimate	SS	df	*F*	*p*
Centroid size	0.051	0.017	1	12.285	**<.001**
Vulnerability	0.011	0.004	1	2.678	**<.01**
Sex	0.032	0.011	1	7.634	**<.001**
Gear	0.020	0.007	1	4.759	**<.001**
Sex: Vulnerability	0.004	0.001	1	0.926	.479
Gear: Vulnerability	0.004	0.001	1	0.944	.505
Residuals	0.878	0.294	211	–	–

Note

Effect sizes were calculated by dividing each covariate's variance by total variance.

**FIGURE 5:**
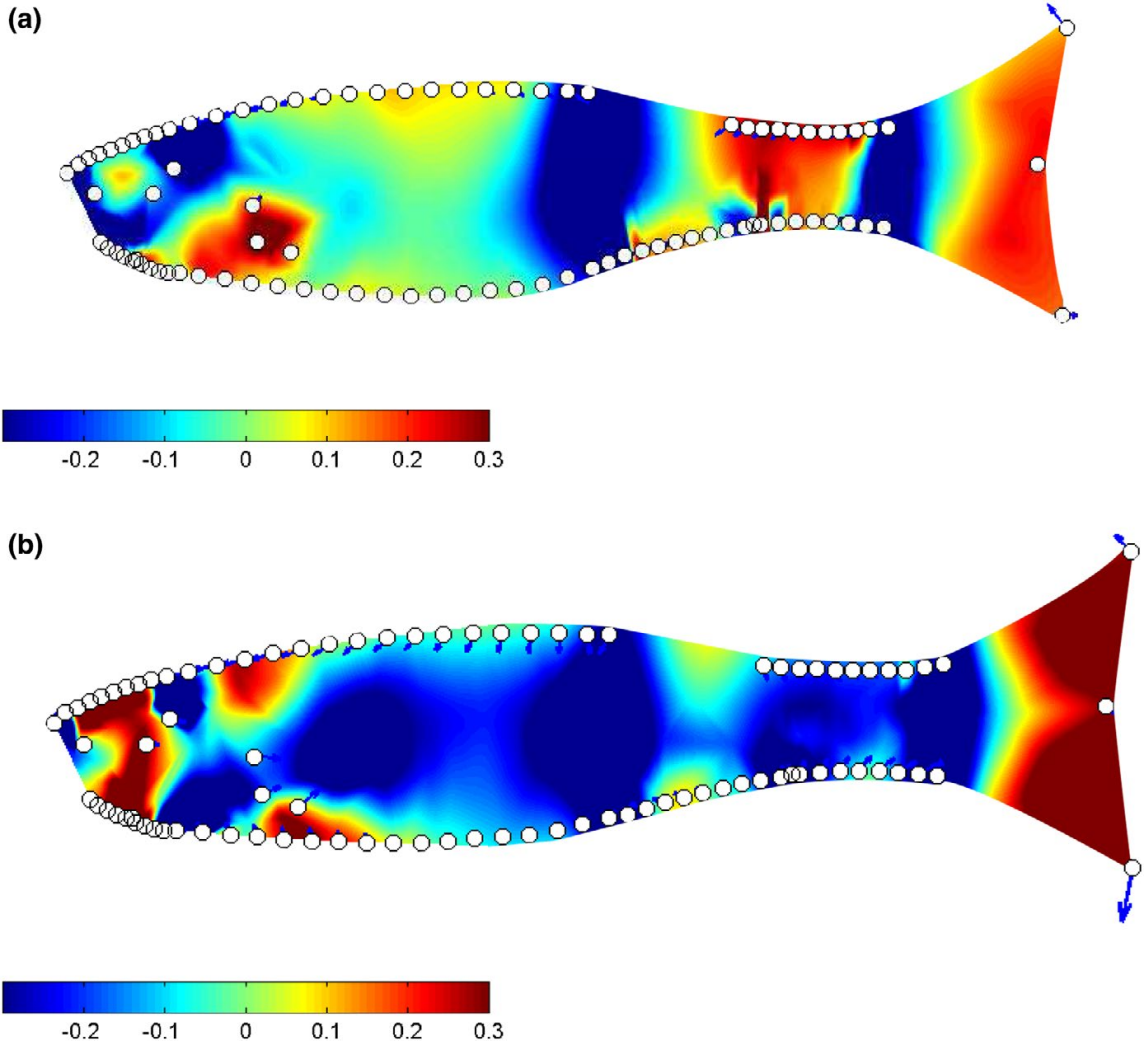
Heat maps of local shape deformation for trawl fish: (a) are female (*n* = 55), and (b) are male (*n* = 56). Red hues are indicative of expansion between the mean shape of high vulnerability fish and the mean shape of low vulnerability fish, blue hues are indicative of contraction, and green hues are approximately invariant regions. Arrows, if present, show the direction of the expansion. For all plots magnification was set at four, and plot resolution at 7,000

Overall, low vulnerability trawl fish were deeper in the body when compared to high vulnerability fish, and had a larger caudal peduncle. In comparison to low vulnerability fish, high vulnerability trawl individuals showed expansion around the posterior insertion of the anal fin, and contraction around the caudal peduncle, this was clear in both sexes. Low vulnerability male fish showed a markedly narrower snout, and the same was not apparent for females (Figure 5). The shape of the caudal fins among vulnerability groups differed significantly, and this difference was especially clear between low and high vulnerability male fish (Figure 5b). The landmarks around the anterior and posterior middle axis of the eye were more widely spaced in high vulnerability trawl fish compared to low vulnerability trawl fish, and this was apparent across both sexes (Figure 5).

**FIGURE 6:**
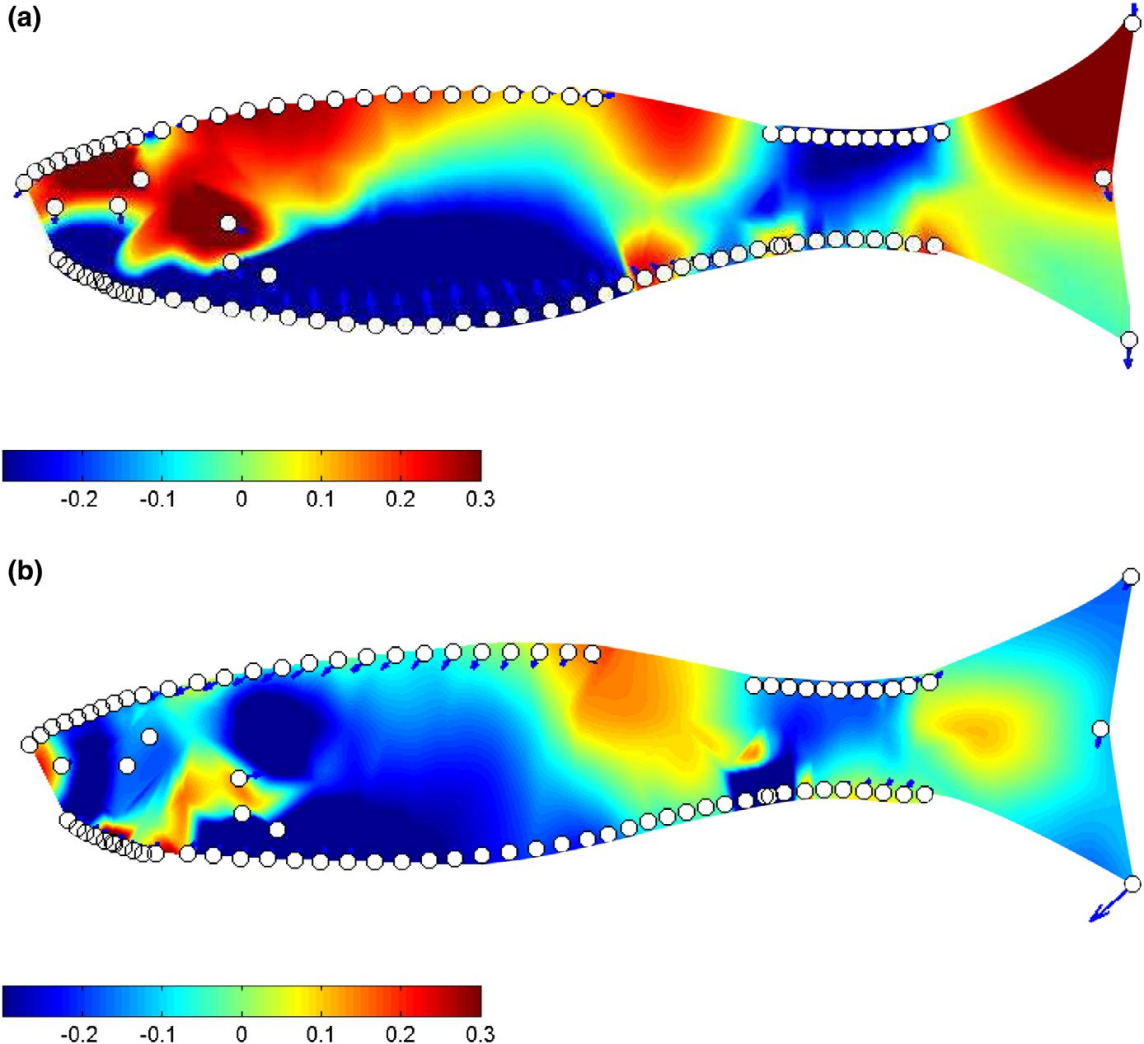
Heat maps of local shape deformation for trap fish: (a) are female (*n* = 44), and (b) are male (*n* = 63). Red hues are indicative of expansion between the mean shape of high vulnerability fish and the mean shape of low vulnerability fish, blue hues are indicative of contraction, and green hues are approximately invariant regions. Arrows, if present, show the direction of the expansion. For all plots magnification was set at four, and plot resolution at 7,000

Similar to trawl fish, low vulnerability trap fish displayed a deeper body, and this general trend was such that at a global level the MANCOVA design indicated no interaction term between vulnerability and gear (Table 1). However, at a finer scale, in contrast to trawl fish, the caudal fin of low vulnerability trap fish was more similar to high vulnerability fish, with no major shape difference being present (Figures 5 and 6). In addition, unlike in trawl fish, where the difference in caudal peduncle shape between high and low vulnerability groups was concentrated at the posterior end of the fish, for trap fish the difference was spread throughout the caudal peduncle region (Figure 6). High vulnerability trap fish were also shallower in the body, particularly around the front of their ventral area, and this was extremely prominent in female fish (Figure 4a,b).

The best fitting linear regression model for caudal fin area included a significant effect of standard length (df = 212, *β* = 2.31, *t* = 10.32, *p* < .001) and gear (df = 213, *β* = −3.82, *t* = −4.07, *p* < .001) (with trap fish showing a smaller caudal fin area). The best fitting model did not include significant interactions, nor did it include vulnerability. The best fitting linear regression model for caudal fin aspect ratio included vulnerability as a fixed effect (df = 213, *β* = −.09, *t* = −1.81, *p* = .069) (indicating that low vulnerability individuals had a lower caudal fin aspect ratio); both interactions and fixed effects of sex and gear were removed from the final model.

Linear discriminant analysis showed clear classification of groupings based on vulnerability, with 75% and 65% of the individuals correctly classified in trawling and trapping groups, respectively.

**FIGURE 7:**
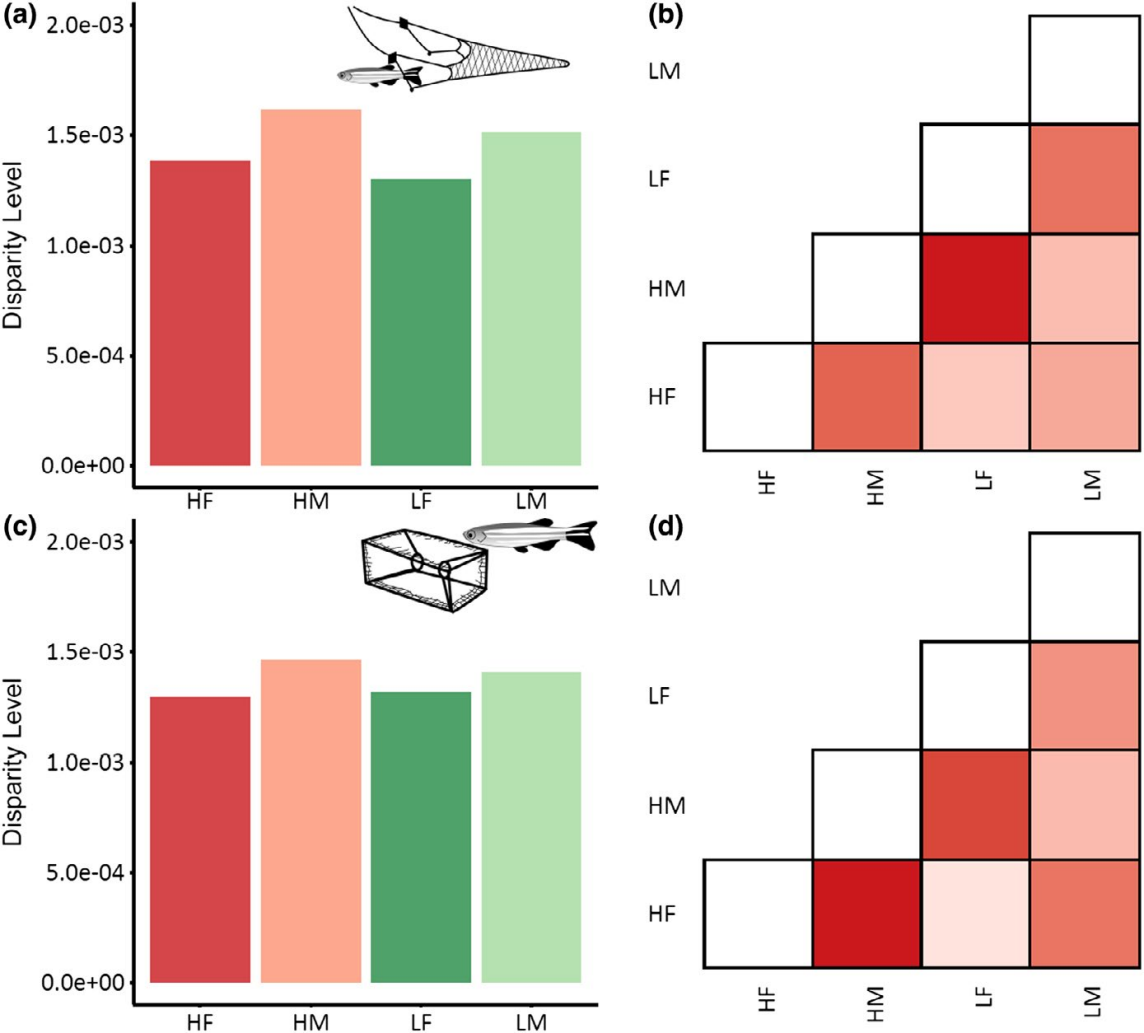
Shape disparity across sex, gear and vulnerability. (a) Shape disparity in trawl fish (*n* = 111) and trap fish (*n* = 107) (c); greens are low vulnerability individuals (LF = Low Female; LM = Low Male) and reds are indicative of high vulnerability individuals (HF = High Female; HM = High Male). Pairwise comparisons for trawl fish (b) and trap fish (d); red hue is indicative of higher disparity and white of lower disparity

Shape disparity level based on Procrustes variance was greatest for high vulnerability male fish across both gear types, while it was consistently lowest for female fish across both gears and vulnerability groups (Figure 7). From pairwise significance testing no significant effects were found in shape disparity (Table S5).

**FIGURE 8:**
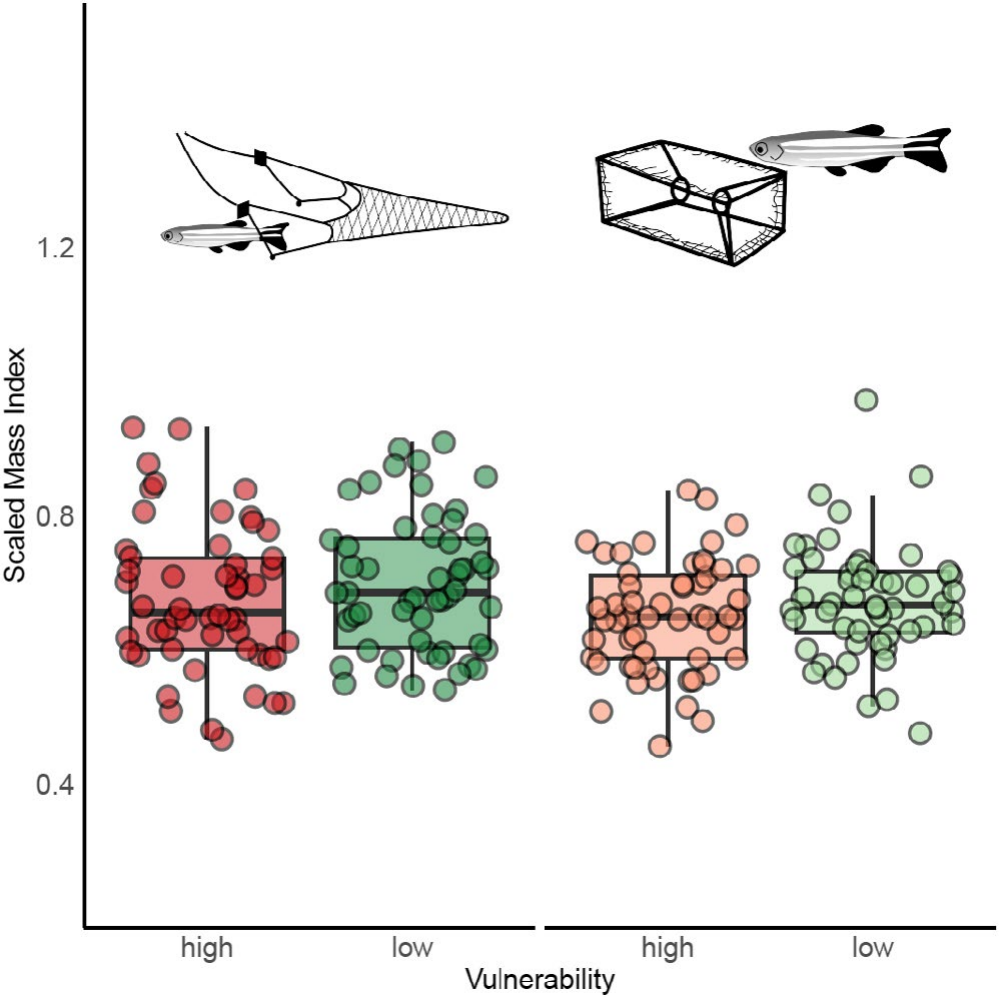
Scaled mass index across gear types, with trawl fish on the left and trap fish on the right of the graph. Each dot is representative of a single fish, greens are low vulnerability individuals and reds are indicative of high vulnerability individuals. The boxplots indicate medians, 25th, and 75th percentile

The best fitting linear regression model for SMI did not include any interactions, but included all fixed terms. Fish that were captured less had a higher SMI (df = 213, *β* = .02, *t* = 1.79, *p* = .08) in both trawl and trap groups (Figure 8; Table S6), although this effect was not significant. SMI did not differ between gears (df = 213, *β* = .02, *t* = 1.53, *p* = .11), but was found to differ between sex (df = 213, *β* = −.08, *t* = −7.07, *p* < .001) with females having a greater SMI than males.

## 5 DISCUSSION

Simulated trawling and trapping both selectively captured zebrafish with distinct morphological phenotypes. Fish with low vulnerability to trawling had a deeper body and a larger caudal peduncle than those with a high vulnerability. Low vulnerability trap fish also displayed a deeper body when compared to high vulnerability trap fish, but differences in shape were centered on the ventral region of the fish. We found a <2% change in shape attributable to fishing vulnerability, and although this seems relatively low, even small phenotypic morphological changes are known to have important adaptive consequences (Smith, 1993). These results are consistent with the hypothesis that commercial fishing in the form of trawling and trapping could impose evolutionary selection pressure on body shape. There was evidence that condition (SMI) was higher in low vulnerability trawl and trap fish, suggesting that fish in better condition may escape trawls and traps more readily. Given that for many fish populations, exploitation rates from fishing are estimated to be far in excess of those of natural predators (Darimont et al., 2015), and that we saw clear evidence both simulated trapping and trawling can select specific body shapes, the results here suggest that there is potential for commercial fishing to influence functional morphology in wild fish populations.

Overall, our simulations point to the fact that both trapping and trawling have the potential to be selective on body shape, with both gear types being more likely to capture shallower‐bodied individuals. The apparent convergence in vulnerable morphological phenotypes across gears could be the result of correlated selection across different traits. Evidence from both zebrafish and other cyprinids that utilize a body/caudal fin mode of swimming indicates that deeper body shapes and deeper caudal peduncles often increase unsteady swim performance (Domenici et al., 2008; Shukla & Bhat, 2017). Therefore, in the context of our trawl simulations, fish with a shallower body shape may have been more prone to being captured owing to having a lower capacity for unsustained swimming. In contrast, in trap simulations, increased captured of shallow‐bodied morphologies may have been related to different traits that covary with shape. For instance, the speed of movement during spontaneous activity has been found to be higher in cyprinids with shallower body shapes (Andersson et al., 2006), which could explain why shallower bodied individuals were captured more often in simulated trapping, as more encounters would mean more chance to be captured. Dimensions of behavior such as boldness may also correlate with body shape and swimming capacity (Hollins et al., 2018). For example, zebrafish that have been selectively bred for boldness have been found to have deeper caudal regions (Kern et al., 2016). This type of relationship between behavioral and morphological characteristics could act together to influence vulnerability to capture. Assuming that passive gears primarily capture the boldest individuals (Arlinghaus et al., 2016), there could be correlated selective capture on morphological traits associated with this type of behavior. In our study, however, it was shallow‐bodied fish that were captured most often in the traps; and as we did not test behavioral phenotypes, it is difficult to establish whether the selection pressure we saw was due to direct selection on morphology from fishing, or a behavioral correlate of morphology. Another possible explanation for deeper bodied fish being less vulnerable to trapping is that shallow‐bodied fish have higher intrinsic hunger levels, exploration rates, or energy demands, and are therefore more willing to enter baited traps (Hansen et al., 2015). A further possibility is that deeper‐bodied fish were less vulnerable to trapping as they found it more difficult to enter the traps. Although trap design in this study was such that all fish could fit into the traps, fish with a larger abdominal diameter may have contacted the entry walls more often and so were perhaps less likely to enter. Given the results of this study, we suggest that further work should aim to link behavior, morphology, and mechanisms of capture during gear encounter.

Although overall selection pressures on shape were similar across the gears tested, there were some morphological differences between fish caught in traps and trawls. Both low vulnerability trawl fish and trap fish were found to have deeper caudal peduncles than their high vulnerability counterparts (see Figure 2), but the divergence in caudal peduncle shape between high and low vulnerability trawl fish occurred primarily at the inception of the caudal fin (Figure 5a,b), while for trap fish the difference seemed to be both weaker and spread throughout the caudal region (Figure 6a,b). In addition to caudal peduncle shape, caudal fin area differed between the two gear types, with trap fish on average having a smaller caudal fin. Both of these morphologies are known to positively influence swimming performance (Blake, 2004; Domenici et al., 2008), and the differences seen between trap and trawl fish could be related to the relative importance of these regions to capture in each of the gear types. Further work addressing how both fin shape and muscle mass throughout the body influences capture vulnerability would benefit future research.

Another subtle difference between the physical selection exerted by the gear types was that trap fish were particularly deep across the abdominal area, while trawl fish were particularly deep in the pre‐anal region. Trapping, in contrast to trawling, principally relies on a behavioral decision by the fish to enter the gear and so it is likely that the suite of traits targeted by trapping differs substantially to that of trawling (Diaz Pauli & Sih, 2017). For instance, behavior (e.g., sociality; boldness) and sensory capacity (e.g., olfaction) are strong candidates for evolutionary selection by traps (Diaz Pauli et al., 2015; Thambithurai et al., 2018). A key difference between passive fishing gears and predators in the wild is that the latter is likely to stimulate an escape response, and therefore may promote selection on specific physiological or morphological adaptations to facilitate this behavior (e.g., deeper body) (Domenici et al., 2008). In contrast, trapping can be described as fully passive (i.e., as far as we know there is no escape response toward a trap when sighted), therefore selection by traps is more likely to target traits associated with exploration or foraging. These differences in the mechanisms of selection and correlations between targeted traits and morphology may be responsible for the subtle differences in morphology observed between fish vulnerable to trapping and trawling, despite the striking similarity we saw in overall vulnerable phenotype.

Previous studies of zebrafish critical swimming performance (*U*_crit_) have indicated that, on average, males have a higher swimming performance threshold as compared to females, with morphological shape accounting for some of this variation (Conradsen & McGuigan, 2015; Leris et al., 2013). The interaction term we included in the global MANCOVA between sex and vulnerability was not significant, indicating that within high and low vulnerability groups, morphology did not differ between sexes. This suggests that although there is an underlying difference in morphology owing to sex (and potential performance), this does not influence the vulnerability of an individual to capture (i.e., the morphologies that make some fish vulnerable are independent of sex). Males showed consistently higher disparity—or degree of variation within a given vulnerability category—than female fish, both for trawl and trap. This could be related to sex‐specific shape dimorphism, as zebrafish females are constantly producing ova with a cycle of approximately 5 days (Eaton & Farley, 1974). At nearly all stages females have an accentuated abdomen which likely homogenizes morphology among females. For males, however, shape may be more directly associated with condition and locomotor ability. High vulnerability fish across gears and between sexes (with exception of female trap fish) showed a higher degree of morphological disparity compared to low vulnerability fish. Overall, however, results suggest that variation in morphological shape traits within high and low vulnerability groups is relatively consistent.

Low vulnerability fish had a higher SMI across both gear types. For trawling, one possible explanation is that fish in better condition are able to escape more often, as these individuals may attain higher swimming performance (Martínez et al., 2004). For trapping, our results were consistent to previous work assessing fishery selection of brown trout (*Salmo trutta*), which were found to have a higher likelihood of capture when in lower condition, owing to increased activity (Härkönen et al., 2014). Presumably the link between condition and increased activity/foraging is higher intrinsic hunger levels (Gotceitas & Godin, 1991). For instance, cod (*Gadus morhua*) in lower physical condition have been found to be more highly attracted to baited traps than those in higher condition, suggesting that intrinsic hunger might be driving capture vulnerability by traps (Huse et al., 1999). Body condition is related to many life‐history traits including size at maturity and fecundity (Marteinsdóttir & Begg, 2002; Morgan, 2004), thus the potential impacts on population dynamics as a result of selection on condition could be considerable. Clearly, further work is needed to elucidate how condition influences capture and selection across different gear types. On the one hand, being in better condition may afford more opportunities for escape owing to higher performance (e.g., higher unsteady swimming); on the other, it may, due to an increased size, maximize the likelihood of entanglement or decrease the likelihood of a fish fitting and escaping through the mesh of a net. Although SMI differed between high and low vulnerability fish, it is difficult to directly compare the relative importance of shape and condition in the capture process, and further work directly addressing this question would be beneficial to the field.

In wild fish populations, predation pressure is known to influence prey fish shape phenotype through selection, typically leading to morphologies that enhance locomotor function: fish with better swimming performance escape more often from predators which either ambush or chase down their prey fish (Heynen et al., 2017; Ingley et al., 2014; Langerhans et al., 2004). Although morphological evolution among prey‐fish will vary according to the specific swimming style they employ, for species that utilize caudal fin propulsion (such as zebrafish), a deepening of the body and caudal peduncle often develops in response to predation (Domenici et al., 2008). There is a lack of evidence pertaining to how zebrafish shape is influenced by natural fish predators, but work that has assessed intraspecific differences in shape that arise as a result of differing environmental conditions suggests that zebrafish with higher swimming performance have a deeper body and caudal peduncle (Shukla & Bhat, 2017). Therefore, we expected that similarly to chase predators, trawling would be more likely to capture individuals with a shallower body shape, and indeed this is what was observed.

In the current study we restricted our simulations to the final stages of capture, possibly limiting any widescale selection occurring on increased encounter rates between gear and fish. For instance, in a natural environment trapping could select fish which have larger home ranges and increased risk‐taking behavior. Typically, shallower and more elongated fish are considered to both engage in longer periods of swimming and having faster cruising swim speeds (Langerhans & David, 2010). This, in theory, should increase the encounter rate between fish and gear, and therefore increase overall selective potential for shallow bodied fish, although this mechanism is impossible to replicate in a laboratory setting. This highlights the importance of combining field‐based approaches, which take into account selection at greater spatial scales, with laboratory approaches that enable an accurate estimation of selection at the point of capture. Morphological responses to predation are complex, and in some cases, local habitat gradients might influence body shape more than predation *per se* (Burns et al., 2009). An additional parameter that could be quantified in future work is the fineness (a measure of fish length relative to its transverse sectional diameter) ratio of fish (Walker et al., 2013). This measure could provide an indication of how important being streamlined is to trawl vulnerability. We did not quantify overall fin area in this study, but maneuverability afforded by larger fins likely also plays a role in vulnerability (Langerhans & David, 2010).

The simulated setup used, as well as the use of zebrafish as a model species, clearly cannot replicate all capture mechanisms and possible outcomes that would be present in a full‐scale trawl scenario. For instance, the trawl used in this experiment was static, and as such the capture dynamics presented to the fish are different to those they may experience in the wild. Nonetheless, the swimming performance of the fish is likely to be a key parameter in both scenarios. For example, in our simulation fish could not fit through the mesh to escape the trawl, although escape underneath the ground gear and sides of the trawl frame (between the trawl and the side walls of the flume) was possible, the location of these escape routes relative to the swim path of the fish may have differed from that of an actual trawl. Still, being able to maintain position ahead of the trawl path for the duration of the trawl is a key determinant of escape from actual trawls, and the trawl simulations used here (Winger et al., 2010). Furthermore, while wild zebrafish are not subjected to the same trawl and trap designs used here, they bear many behavioral similarities to species that are targeted by these gears, including their social behavior and swimming mode. One thing we didn't consider that could vary from how wild fish populations respond to selective harvest is how dominance hierarchies established during the experiment (owing to fish being kept in tanks together) influenced capture. The only work that has addressed this point has found that fish trawled in familiar shoals (i.e., able to establish more stringent hierarchies) displayed a higher propensity for escape to trawls than fish tested in unfamiliar shoals (Hollins et al., 2019). Further work is needed to elucidate the link between dominance and capture in active and passive fishing gears. Although caution should be used in generalizing these results to wild populations, scaled down simulations with surrogate species provide a key starting point to examine fishery selection on traits that up to now have received little attention (Alós et al., 2014; Lennox et al., 2017). Repeated fishing trials of the same individuals are extremely challenging in full‐scale fisheries, and a range of additional factors may interfere with the ability to detect selection on morphological traits. The results here can therefore inform the design of logistically challenging and expensive studies to examine how these effects carry over into actual fisheries.

## 6 SUMMARY

In conclusion, our results suggest that both trawling and trapping can select for specific body morphologies. Fish that readily escape trawls showed morphological characteristics which reflect enhanced burst‐swimming performance (deeper body, larger caudal peduncle, narrow snout), partly matching natural evolution of prey fish faced with non‐human wild predators (Domenici et al., 2008; Langerhans et al., 2004). Both gears captured shallow shaped fish more often, although some differences were noticeable: e.g., low vulnerability trawl fish were deepest in the pre‐anal region and their caudal peduncle was thickest just before the inception of the caudal fin, while low vulnerability trap fish were deepest in the abdominal region and had a deeper caudal peduncle along the length of the tail. Clearly, the selective processes seen in this study may result in direct morphological change, but changes to life‐history and fitness owing to correlated selection on morphology are also possible. An interesting avenue for future studies would be to understand what the role of intrinsic states such as hunger has on fish capture, especially in static gears where behavior is predicted to be more important. These results contribute to an increasing body of evidence pointing to fishing having the potential to select on a number of traits beside size (Alós et al., 2016; Biro & Post, 2008; Diaz Pauli & Sih, 2017; Killen et al., 2015). Further work is required to establish whether similar selective processes on morphology are occurring in wild commercially exploited fish populations, and whether these warrant concern amid other evolutionary pressures.

## CONFLICT OF INTEREST

The authors declare no competing or financial interests.

## AUTHOR CONTRIBUTIONS

**Davide Thambithurai:** Conceptualization (lead); data curation (lead); formal analysis (lead); investigation (equal); methodology (equal); visualization (lead); writing – original draft (lead); writing – review and editing (lead). **Anita Rácz:** Resources (equal); writing – review and editing (supporting). **Jan Lindstrom:** Conceptualization (supporting); supervision (supporting); writing – review and editing (supporting). **Kevin J. Parsons:** Conceptualization (supporting); supervision (supporting); writing – review and editing (supporting). **Shaun Killen:** Conceptualization (supporting); funding acquisition (lead); methodology (supporting); project administration (supporting); supervision (lead); writing – review and editing (supporting).

### OPEN RESEARCH BADGES

This article has earned an Open Data Badge for making publicly available the digitally‐shareable data necessary to reproduce the reported results. The data is available at https://datadryad.org/stash/share/QrA14DhtfuaprEjvbgUuK2W9kfSg1uBu5yt7MkGSlpA.

## Supporting information

Appendix S1

Video S3

Supplementary Material

## Data Availability

Data available from the Dryad Digital Repository: https://doi.org/10.5061/dryad.pk0p2ngq2.
