# Supplementary material for: Simulated trapping and trawling exert similar selection on fish morphology
Source: Ecol Evol. 2022 Feb 12;12(2):e8596. doi: 10.1002/ece3.8596 (PMC8840878; doi:10.1002/ece3.8596)
Supplement: Supplementary file 1 — Appendix S1 [file ECE3-12-e8596-s001.docx]

**Supplementary Materials**

**Table S1.** Standard length ranges, mass ranges and sex ratios for fish used in the experiment. Note that a few fish are missing from this table as mass/lengths were missing for them.

| **-** | **Standard length range (mm)** | **Mass range (g)** | **High group sex (f:m)** | | **Low group sex (f:m)** | |
| --- | --- | --- | --- | --- | --- | --- |
| Trap group | 24.7-40.3 | 0.27-1.38 | 23 | 32 | 21 | 31 |
| Trawl group | 25.5-38.6 | 0.27-1.14 | 26 | 28 | 29 | 28 |


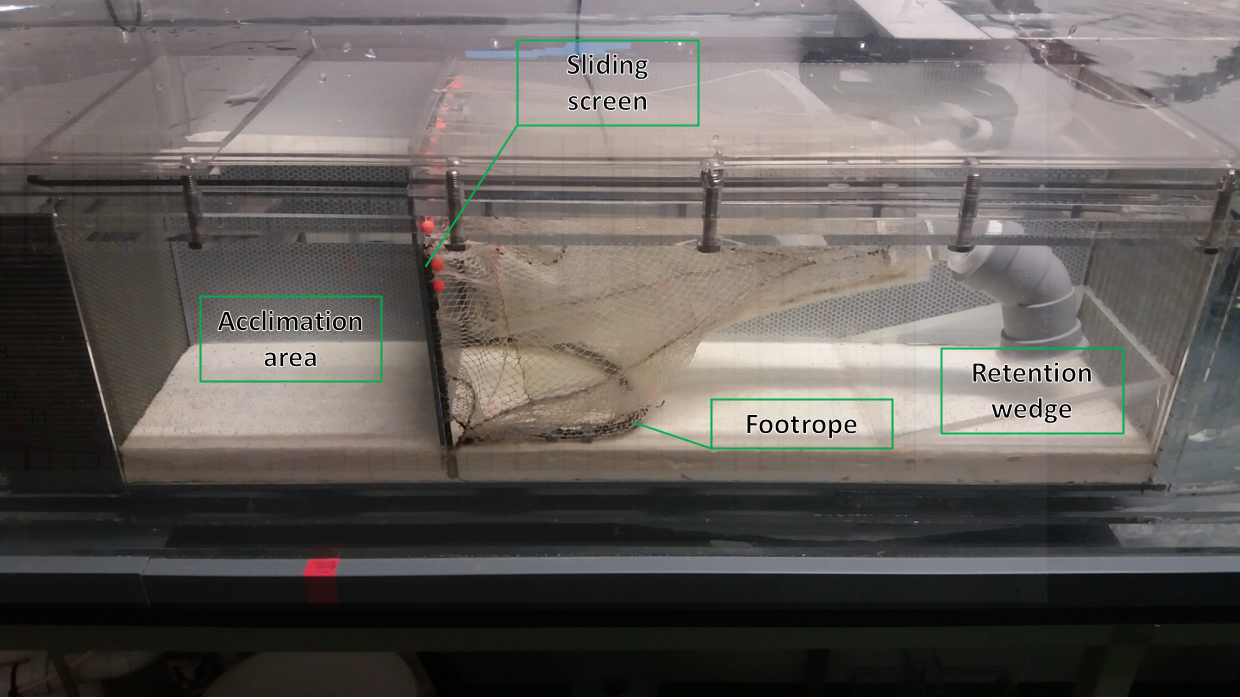


**Figure S2.** Trawl setup used in experiment.

**Movie S3.** Movie clip showing trapping experiment. Lid is lifted from acclimation chamber and fish begin to explore the trapping arena. Three baited traps can be seen, one in the foreground and two in the background. The location of traps and plants was randomized at the beginning of the trial. At 00:00:20 a fish can be seen entering the trap in the rightmost trap.

**Script S4.** Supporting R scripts. Note that main figures are not reproduced here.

**Table S5.** P-values obtained from the morphol.disparity function (geomorph package) representative of pairwise differences between vulnerability groups for trawl and trap fish (A and B respectively).

| **(A)** | **High.female** | **High.male** | **Low.female** | **Low.male** |
| --- | --- | --- | --- | --- |
| **High.female** | 1 | - | - | - |
| **High.male** | 0.1637836 | 1 | - | - |
| **Low.female** | 0.6200380 | 0.05019498 | 1 | - |
| **Low.male** | 0.4297570 | 0.52934707 | 0.18138186 | 1 |
| **(B)** | **High.female** | **High.male** | **Low.female** | **Low.male** |
| **High.female** | 1 | - | - | - |
| **High.male** | 0.2737726 | 1 | - | - |
| **Low.female** | 0.8900110 | 0.3670633 | 1 | - |
| **Low.male** | 0.4795520 | 0.6913309 | 0.5874413 | 1 |

**Table S6.** Mean ± standard deviation of scaled mass index across gear and treatment groups

| **-** | **High Vulnerability** | **Low Vulnerability** |
| --- | --- | --- |
| Trap group | 0.65 ± 0.08 | 0.67 ± 0.09 |
| Trawl group | 0.68 ± 0.11 | 0.7 ± 0.1 |
